# Latency-Aware Benchmarking of Large Language Models for Natural-Language Robot Navigation in ROS 2

**DOI:** 10.3390/s26020608

**Published:** 2026-01-16

**Authors:** Murat Das, Zawar Hussain, Muhammad Nawaz

**Affiliations:** 1 Sydney Polytechnic Institute, Sydney, NSW 2000, Australia; dasmuratr@gmail.com (M.D.); muhammad.nawaz@student.uts.edu.au (M.N.); 2 School of Computing, Macquarie University, Sydney, NSW 2109, Australia; 3 School of Computer Science, University of Technology Sydney, Sydney, NSW 2007, Australia

**Keywords:** large language models, robot navigation, ROS 2, natural-language interfaces, latency benchmarking, Navigation 2 (Nav2), local planners, human–robot interaction

## Abstract

A growing challenge in mobile robotics is the reliance on complex graphical interfaces and rigid control pipelines, which limit accessibility for non-expert users. This work introduces a latency-aware benchmarking framework that enables natural-language robot navigation by integrating multiple Large Language Models (LLMs) with the Robot Operating System 2 (ROS 2) Navigation 2 (Nav2) stack. The system allows robots to interpret and act upon free-form text instructions, replacing traditional Human–Machine Interfaces (HMIs) with conversational interaction. Using a simulated TurtleBot4 platform in Gazebo Fortress, we benchmarked a diverse set of contemporary LLMs, including GPT-3.5, GPT-4, GPT-5, Claude 3.7, Gemini 2.5, Mistral-7B Instruct, DeepSeek-R1, and LLaMA-3.3-70B, across three local planners, namely Dynamic Window Approach (DWB), Timed Elastic Band (TEB), and Regulated Pure Pursuit (RPP). The framework measures end-to-end response latency, instruction-parsing accuracy, path quality, and task success rate in standardised indoor scenarios. The results show that there are clear trade-offs between latency and accuracy, where smaller models respond quickly but have less spatial reasoning, while larger models have more consistent navigation intent but take longer to respond. The proposed framework is the first reproducible multi-LLM system with multi-planner evaluations within ROS 2, supporting the development of intuitive and latency-efficient natural-language interfaces for robot navigation.

## 1. Introduction

Natural-language dialogue has become an exciting means to control mobile robots without using traditional visual interfaces or strict human–machine interfaces (HMIs). People who want to use traditional robot navigation pipelines usually need to know how to use complicated software programs and structured input layouts. These challenges make it harder for non-experts to use the technology and make it harder to use mobile robots in daily life. Recent improvements in Large Language Models (LLMs) have shown great promise for turning free-form human instructions into logical robotic actions, making it easier for people to interact with robots [1,2,3]. Consequently, in robotics, integrating LLMs for navigation and spatial reasoning has become an active research area. Several works demonstrate that LLMs can interpret ambiguous commands, infer spatial relations, and generate high-level action plans [4,5,6]. Others show that multimodal or vision–language models can further improve perception-driven navigation [7,8].

Despite substantial progress, vision–language navigation remains difficult because high-level natural-language instructions must be translated into concrete perceptual cues and executable navigation goals. In many real-world scenarios, users tend to express abstract objectives (e.g., “go to the meeting room”) rather than detailed motion trajectories. As shown in prior work on language-assisted vision-based navigation, this places a significant burden on the system to resolve ambiguities between the linguistic intent, visual perception, and motion planning [9]. These challenges become even more pronounced when classical local planners are required to operate on goals inferred through semantic reasoning rather than explicitly defined geometric targets. In practical deployments, extracting reliable navigation goals from language is further complicated by perceptual uncertainty, sensor noise, and incomplete or noisy spatial information. Studies on camera-based perception indicate that vision-only systems frequently struggle with accurate depth estimation and object localisation, which can introduce cascading errors in downstream navigation and planning modules [10]. For these reasons, isolating the interaction between language understanding and navigation planning within a simulated environment provides a controlled and informative first step, allowing the effects of language-derived goals to be evaluated without the confounding influence of real-world perceptual limitations.

Despite this rapid progress, most reported systems still lack standardised benchmarking frameworks, especially for evaluating latency, an essential factor in time-critical robotic tasks. Latency is especially important, because LLM-based processes add extra inference delays that are not there in traditional ROS 2 navigation systems. Research has highlighted latency as a key limitation limiting LLM-based real-time control, with lags permeating instruction processing, action generation, and planner implementation [11]. On the other hand, benchmarking efforts in LLM research typically focus on computational performance [12,13,14] rather than embodied robotic interaction or navigation accuracy. Meanwhile, existing robotics benchmarking frameworks—such as Arena 4.0 [15], ROS2-based evaluation platforms [16], and planning-system benchmarks [17,18]—evaluate only classical planners and do not incorporate natural-language processing components. These gaps reveal that there is a need for a unified framework that can measure (i) how well LLMs parse instructions, (ii) how accurately planners navigate, and (iii) how long it takes for a response to get from one end to the other. This kind of framework would allow comparing different LLMs, planners, and model sizes for implementing conversational robots for production.

This work addresses these challenges by introducing a latency-aware benchmarking framework that integrates multiple LLMs with the ROS 2 Navigation 2 (Nav2) stack. Using a simulated TurtleBot4 platform in Gazebo Fortress, we measure the LLM response latency, instruction-parsing correctness, trajectory quality, and task success rate across three widely used local planners: Dynamic Window Approach (DWB), Timed Elastic Band (TEB), and Regulated Pure Pursuit (RPP). We benchmark a diverse set of state-of-the-art LLMs, including GPT-4, GPT-5, Claude 3.7, Gemini 2.5, Mistral, DeepSeek, and LLaMA, to quantify their trade-offs between speed and spatial reasoning ability.

The main contributions of this research work are as follows:

- We propose the first unified multi-LLM system with multi-planner benchmarking frameworks for ROS 2 navigation that uses natural language instructions.
- We introduce a reproducible latency-evaluation pipeline that measures the time it takes to parse instructions, extract intent, and generate actions.
- We produce a technical analysis on how well DWB, TEB, and RPP planners can navigate in identical situations, showing the pros and cons of each planner when following LLM-generated commands.
- We demonstrate how the LLM size, response time, and navigation accuracy are related, and we find clear trade-offs between latency and performance.

By quantifying both the linguistic and physical navigation capabilities of contemporary LLMs, this work contributes a foundational step toward practical, conversational, and latency-efficient mobile robots.

## 2. Materials and Methods

### 2.1. Research Design and Rationale

This study applies an empirical simulation-driven research design to assess the integration of LLMs with the ROS 2 Navigation 2 (Nav2) framework for natural-language robot navigation. Simulation is preferred over real-world deployment due to its safety, reproducibility, and resource efficiency. Unlike physical experiments, which are subject to hardware wear, environmental noise, and high setup costs, simulation allows controlled trials with repeatable conditions. Reinitialising the Gazebo world before each trial ensures that the robot starts from identical initial states, minimising confounding factors. A simulation-centric procedure enhances experimental scaling by facilitating large-scale repeatable trials devoid of the technical constraints associated with battery recharging, hardware degradation, and platform reset intervals. This design choice is consistent with established robotics practice, in which high-fidelity simulation functions as the primary validation environment prior to constrained real-world deployment. While simulation cannot perfectly replicate reality, it captures core challenges of localisation, path planning, and control, and prior work has shown that insights from simulation, such as latency and parsing robustness, generalise well to real deployments.

The design is comparative in nature. Multiple commercial and open-source LLMs are tested on their ability to interpret natural-language commands into structured navigation goals. After that, various Nav2 planners run these results, which lets one compare their performance. To answer those questions, metrics like delay, interpretation accuracy, path accuracy, and effectiveness are recorded. The study addresses a gap in research by benchmarking across both dimensions, language comprehension and motion planning, rather than examining them in isolation.

Importantly, the primary aim of this work is not to validate physical robot behaviour, but to evaluate whether large language models can reliably replace traditional task-specific human–machine interfaces for navigation by interpreting free-form natural-language commands. From this perspective, simulation provides an appropriate and sufficient evaluation setting, as it allows the end-to-end language-to-navigation pipeline to be assessed under controlled repeatable conditions. Conducting the study in simulation ensures that observed differences in latency, parsing robustness, and planner behaviour arise from the LLM–planner integration itself rather than from uncontrolled hardware or environmental variability. Real-world deployment and physical validation are therefore considered a natural extension of this framework, rather than a prerequisite for its core contribution.

### 2.2. System Workflow

**Figure 1:**
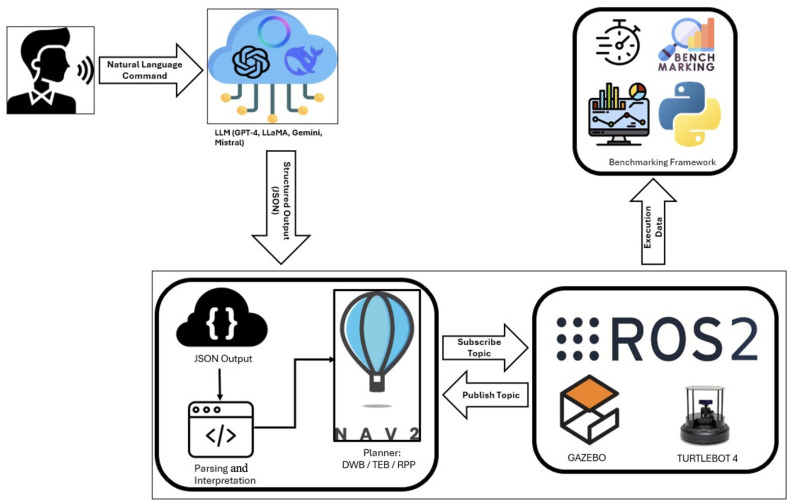
LLM-guided navigation pipeline integrating natural-language commands, LLM inference, JSON parsing, Nav2 planning, Gazebo simulation, and the benchmarking framework.

Figure 1 shows the entire procedure. The pipeline comprises six distinct stages connected through a modular architecture designed for reproducibility.

1. **User command input:** A natural-language instruction such as “Bring me a bottle of water from the table” is issued. This step represents the human–robot interaction (HRI) interface.
2. **LLM processing:** The instruction is forwarded to an LLM via an API call. The evaluated models include GPT, Claude, Gemini, Mistral, DeepSeek, and LLaMA. Each of the models is provided a template that limits the schema and encourages responses in JavaScript Object Notation (JSON) form.
3. **Parsing and validation:** A tradition ROS 2 node checks the response towards a JSON structure that has already been set up. If details are missing or not in the right syntax, the system will try again up to three times before logging an error. This helps make sure that the output looks the same across the various models.
4. **Planner execution:** The structured navigation goal (goal_x, goal_y) is passed to Nav2. Local planners such as DWB, TEB, and RPP convert this goal into feasible trajectories.
5. **Simulation execution:** Gazebo Fortress provides a physics-based environment in which the TurtleBot4 executes the trajectory, navigating around obstacles and reacting to dynamics.
6. **Benchmarking and logging:** A benchmarking framework keeps track of the latency, query accuracy, trend metrics, and whether an outcome was successful or failed. We put all the data into CSV and JSONL files, so we can look at it later.

The system’s modular structure enables systematic and controlled experimentation, as individual components such as the large language model, local planner, and simulation environment can be modified or substituted without affecting the rest of the framework. All interactions are fully recorded, and intermediate artefacts, including structured JSON outputs and execution logs, are stored to ensure that the evaluation process is traceable and repeatable. This design supports fair comparison across different LLM–planner combinations while minimising confounding factors.

To further support reproducibility and open research, the complete implementation—including prompt templates, schema definitions, configuration files, and full experiment logs (CSV/JSONL)—is publicly available in the project’s GitHub (version v0.1) accessed on 5 December 2025 repository at https://github.com/dasmurat/ros2_capstone_SPI.

### 2.3. Simulation Environment

#### 2.3.1. ROS 2 Middleware

ROS 2 is the middleware that makes perception, planning, and control work together. It has a publish-subscribe architecture that lets nodes share data through topics and services. This makes it easy for different modules to work together. Compared to ROS 1, ROS 2 adds real-time communication, quality-of-service (QoS) policies, and multi-robot support, which are crucial for reproducible benchmarking tasks where latency and planner comparisons must be fair and consistent across trials.

#### 2.3.2. Gazebo Fortress Simulator

**Figure 2:**
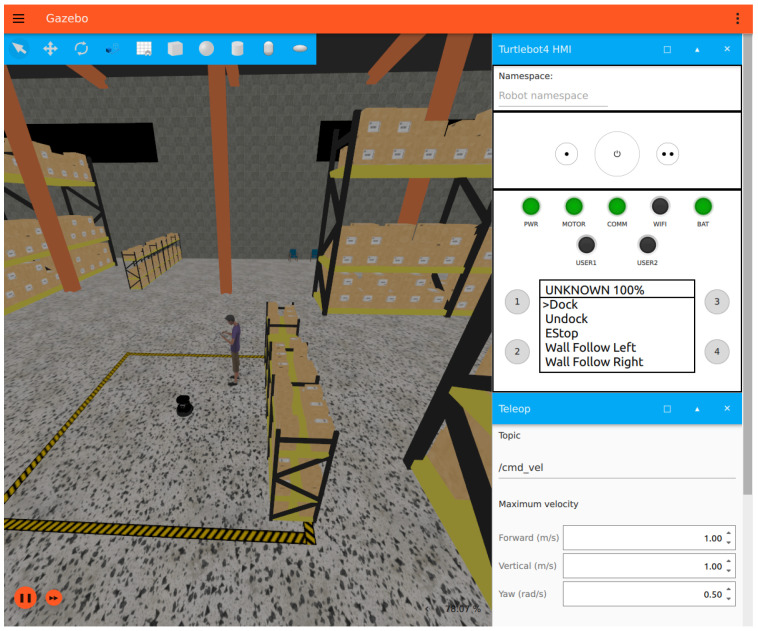
Gazebo Fortress simulation world used for LLM-driven navigation experiments.

Gazebo Fortress was selected as the simulator due to its tight integration with ROS 2 and support for realistic physics. Simulation avoids hardware risk, battery downtime, and environmental noise while still capturing the challenges of localisation and motion planning. The simulator is reset before each trial to ensure identical starting conditions, avoiding bias from hidden factors such as pose drift or accumulated map errors. This reset-based protocol also allows hundreds of trials to be executed consistently, see Figure 2.

#### 2.3.3. TurtleBot4 Platform

**Figure 3:**
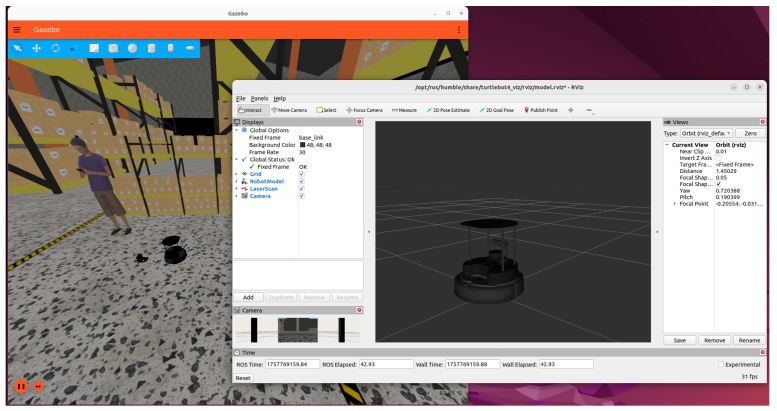
TurtleBot4 model configured in Gazebo Fortress.

The simulated framework is a TurtleBot4 mobile robot model. TurtleBot4 has a 2D LiDAR for finding obstacles, an inertial measurement unit (IMU) for figuring out where it is, and wheel encoders for figuring out how far it has travelled. These sensors represent a typical configuration for service robots. In this study, TurtleBot4 is combined with SLAM Toolbox for mapping, AMCL for localisation, and Nav2 costmaps for dynamic obstacle avoidance, providing a realistic and reproducible setup, see Figure 3.

### 2.4. Nav2 Stack and Planner Architecture

**Figure 4:**
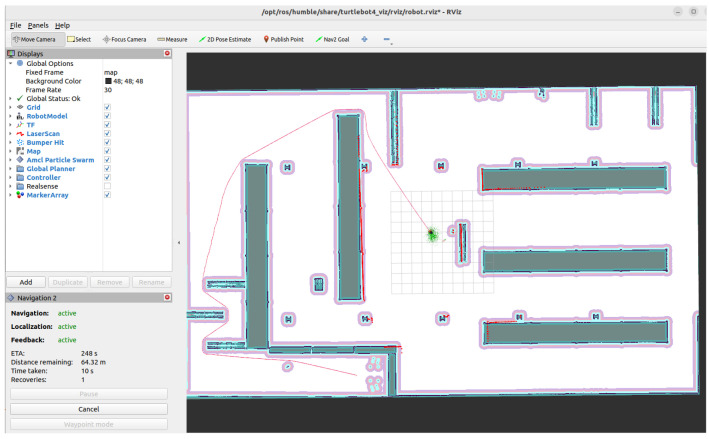
Nav2 local planner architecture and integration with the LLM-driven navigation pipeline.

Rviz2 is used to visualise map data, robot localisation, global plans, and local trajectories, facilitating debugging and validation of the navigation pipeline. Navigation is handled by the Navigation 2 (Nav2) stack, which modularises navigation into global planning, local planning, costmaps, and behaviour trees. Costmaps combine static maps with live sensor input, enabling the robot to respond dynamically to changes in the environment. Nav2 supports multiple local planners within a unified framework, allowing direct comparison under identical conditions, see Figure 4.

Three local planners are evaluated:

- **Dynamic Window Approach (DWB):** a velocity-sampling method that generates candidate commands within dynamic limits and scores them on obstacle clearance, goal progress, and alignment.
- **Timed Elastic Band (TEB):** an optimisation-based planner that adjusts a sequence of poses (the “elastic band”) to minimise cost functions related to smoothness, feasibility, and obstacle clearance.
- **Regulated Pure Pursuit (RPP):** a geometric pursuit method, where the robot tracks a look-ahead point on the path; it is simple and robust, but can struggle in highly dynamic environments.

Parameter tuning is necessary to ensure a fair comparison. Table 1 shows a summary of the representative values employed in the current research. The default Nav2 settings were used as a starting point, and changes were made to find a balance between safety, efficiency, and reproducibility.

### 2.5. Natural-Language Command Set

Natural-language commands form the entry point of the navigation pipeline. Instead of relying on structured coordinates or predefined waypoints, the robot receives free-form textual instructions such as “Go to the docking station” or “Fetch the bottle of water from the table.” These commands are closer to how non-expert users naturally communicate with robots, making the evaluation more realistic for HRI [3,4]. There are two reasons why instructions are given as written content instead of speaking. First, text input removes mistakes that can happen with automatic speech recognition, making sure that the benchmarking only tests LLM reasoning and not transcription precision. Second, instructions that are based on text make it easier to reproduce the results. One identical set of commands can be used in numerous trials without any changes to tone, accent, or noise surrounding [19].

Commands are grouped into three complexity levels:

- **Simple commands** involve direct navigation to a clearly defined destination (e.g., “Go to the docking station”). These establish a baseline for reliability in mapping a single instruction to a valid goal.
- **Moderate commands** add semantic grounding (e.g., “Fetch the bottle of water from the table”), requiring the LLM to associate objects or named locations with navigation goals, even though perception is abstracted in this simulation.
- **Complex commands** require multi-step reasoning (e.g., “Move to the shelf near the entry and then return to the table”), demanding sequential goals and correct execution order.

This categorisation ensures ecological validity and provides a structured way to test how different LLMs handle increasing levels of ambiguity and reasoning difficulty.

### 2.6. LLM Interface and Parsing

#### 2.6.1. Models Evaluated

Six LLM families are evaluated:

- **GPT (OpenAI)**: large language model family used for reasoning and conversation-based navigation tasks; specific evaluated variants are listed in Table 2 [11].
- **Claude (Anthropic)**: highlights safety and trustable reasoning, best suitable for safety-critical HRI settings.
- **Gemini (Google DeepMind)**: a multimodal model capable of dealing text and vision, which is great for future extensions requiring perception [4].
- **Mistral (Mistral AI)**: lightweight, open-source, and latency-optimised; deployable on constrained hardware.
- **DeepSeek**: cost-efficient open-source model appropriate for large fleets or cost-sensitive deployments.
- **LLaMA (Meta), executed via Groq**: open-source model run on Groq accelerators, providing low-latency inference important in dynamic navigation tasks [20].

While this section describes the supported LLM families at a conceptual level, the exact model variants used for experimental evaluation (e.g., GPT-5, GPT-5 Chat, Gemini-2.5 Pro) are listed in Table 2.

#### 2.6.2. Schema-Constrained Outputs

All models are prompted to return responses in a JSON object containing the fields {intent, goal_x, goal_y, note}. In practice, models differ in their reliability: larger models such as GPT and Claude rarely deviate from the schema, while smaller open-source models occasionally produce malformed outputs, including missing fields, extra natural-language commentary, or syntax errors.

To solve this, a schema validation module is implemented in the ROS 2 node. Each model output is parsed and validated; if invalid, the system automatically retries using the exact prompt. Retries are capped at three attempts to balance fairness and efficiency: unlimited retries could mask poor behaviour, whereas too few retries risk discarding recoverable errors. Responses that remain invalid after three attempts are logged as failures and excluded from planner execution. This mechanism standardises the comparison across models and provides a dataset of failure cases for further analysis.

### 2.7. Benchmarking Design

#### 2.7.1. Evaluation Metrics

To benchmark both language understanding and navigation performance, the following metrics are collected:

- **Latency:** Wall-clock time between issuing a command and receiving a valid structured output, including LLM inference and parsing. High latency can negatively impact safety and user experience in dynamic environments [11].
- **Parsing accuracy:** Percentage of LLM responses that pass JSON schema validation without exceeding the retry limit, indicating the robustness of structured output generation [3].
- **Path efficiency:** Ratio of the actual executed path length to the shortest feasible path between the start and goal. The shortest feasible path is computed using the static occupancy grid map available to the Nav2 stack and serves as a consistent baseline reference rather than an optimal planner solution. This metric reflects the navigation efficiency in terms of the travel distance, energy consumption, and task duration.
- **Success rate:** Proportion of trials completed without collision, timeout, or parsing failure, reflecting the overall reliability of the LLM–planner combination.
- **Cost per command:** Monetary cost of API-based LLM calls, calculated from token usage logs. This metric is relevant for large-scale or cost-sensitive deployments [21].

#### 2.7.2. Experimental Procedure

Each trial follows a consistent five-step procedure:

1. Reset the simulation to a baseline state using a Gazebo world reset.
2. Issue a randomly sampled natural-language command from the defined command set to the LLM node.
3. Parse the JSON output and forward valid goals to the selected Nav2 planner.
4. Execute the trajectory in Gazebo with the TurtleBot4 platform.
5. Log latency, success or failure, path metrics, and token usage to CSV and JSONL files.

A minimum of 30 trials are conducted for each LLM–planner combination. This provides sufficient samples for averaging and estimating variability while remaining feasible within computational and time constraints. Commands from simple, moderate, and complex categories are randomly interleaved to prevent overfitting to a single command type and to ensure that the results reflect the performance across the full spectrum of task difficulty. Directory structures are organised into timestamped subfolders containing raw LLM outputs, parsed JSON responses, and performance metrics. This design supports open-science practices by enabling other researchers to inspect, replicate, or extend the benchmarking framework.

### 2.8. Ethics and Reproducibility

The study does not involve human participants or sensitive datasets. Nevertheless, several principles are followed:

- **Transparency:** use of commercial APIs complies with provider terms, and model versions are documented.
- **Reproducibility:** code, configuration files, and experiment scripts are version-controlled using Git.
- **Open science:** datasets and configuration details are structured so they can be shared alongside the manuscript.
- **Resilience:** inclusion of open-source models mitigates reliance on single commercial vendors, although trade-offs in performance are acknowledged.

## 3. Results

This section presents the experimental outcomes of the proposed latency-aware benchmarking framework. The results evaluate (i) LLM inference latency, (ii) parsing and task success rate, (iii) token usage efficiency, (iv) planner-level navigation performance, and (v) qualitative behaviour in simulation.

### 3.1. Overall Performance Overview

Across more than 900 trials, all models successfully integrated with the ROS 2 Nav2 pipeline. Lightweight LLMs produced the lowest latency, while larger reasoning models delivered more consistent accuracy at higher computational cost. The planner performance varied depending on trajectory complexity, with TEB providing the smoothest paths and DWB offering the fastest execution.

### 3.2. LLM Latency Performance

**Figure 5:**
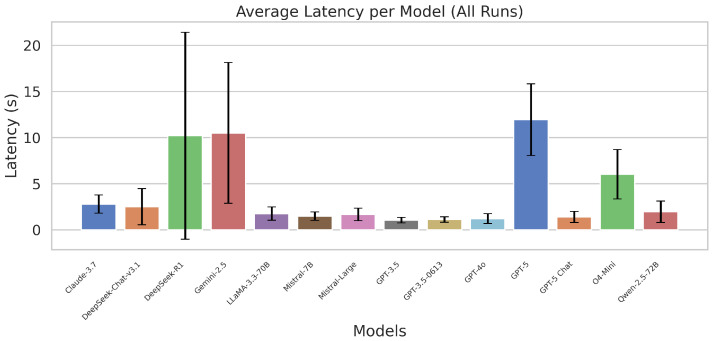
Combined round-trip latency for all evaluated LLMs across simple, moderate, and complex commands.

Figure 5 shows the aggregated inference latency across all LLMs. Models such as GPT-3.5, GPT-4o, and LLaMA-70B on Groq consistently achieved sub-2-second response times, whereas premium reasoning models (GPT-5, DeepSeek-R1, Gemini-2.5) exhibited delays above 10 s.

In addition to the aggregated latency visualisation, Table 2 reports a quantitative summary of LLM performance across all experimental runs. The table includes the mean latency, standard deviation, number of trials, task success rate, and token usage statistics for each evaluated model.

**Table 2:** Combined performance summary across all experimental runs.

Model	Avg Latency (s)	Std Latency (s)	N	Success (%)	Tokens In	Tokens Out	Total Tokens
anthropic/claude-3.7-sonnet	2.712	0.552	250	100.0	90.8	34.3	125.0
deepseek/deepseek-chat-v3.1	2.139	1.704	200	99.5	83.8	32.6	116.4
deepseek/deepseek-r1	9.567	9.087	250	100.0	84.6	356.0	440.6
google/gemini-2.5-pro	9.850	5.455	250	100.0	77.9	773.3	851.2
meta-llama/llama-3.3-70b-instruct	1.818	0.754	250	99.6	92.8	25.3	118.1
mistralai/mistral-7b-instruct	1.505	0.614	250	94.0	93.8	35.8	129.6
mistralai/mistral-large-2411	1.703	0.718	250	100.0	92.8	50.9	143.7
openai/gpt-3.5-turbo	0.968	0.234	200	99.5	87.8	22.1	109.9
openai/gpt-3.5-turbo-0613	1.126	0.219	200	100.0	88.2	22.1	110.3
openai/gpt-4o	1.188	0.312	200	100.0	88.1	26.1	114.2
openai/gpt-5	12.136	3.799	250	100.0	87.0	516.1	603.1
openai/gpt-5-chat	1.272	0.328	200	100.0	88.1	29.1	117.2
openai/o4-mini	5.830	2.699	200	100.0	87.1	307.0	394.1
qwen/qwen-2.5-72b-instruct	2.419	1.599	250	100.0	90.1	28.2	118.4

The reported standard deviation values highlight differences in the latency stability across models. The lightweight and latency-optimised models (e.g., GPT-3.5, GPT-4o, and LLaMA-70B on Groq) exhibit consistently lower variance, indicating predictable response times suitable for time-sensitive applications. In contrast, larger reasoning-oriented models (e.g., GPT-5, DeepSeek-R1, and Gemini-2.5 Pro) show substantially higher variance, reflecting long-tailed latency distributions observed in Figure 5.

The number of evaluated trials (*N*) varies slightly across models, because not all models were tested with identical command counts at each complexity level. As described in Section 2.5, natural-language commands are grouped into simple, moderate, and complex categories, and some models were evaluated with additional or extended test scenarios to better characterise their behaviour under higher reasoning complexity. This design choice prioritises the structured coverage of command complexity over enforcing a fixed trial count, while maintaining fair comparison within each complexity category.

The token usage statistics further illustrate the trade-off between latency and computational cost. Models with stronger reasoning capabilities generally require significantly higher token counts per command, which has direct implications for cost-sensitive or large-scale deployments. Despite these differences, the task success rates remain nearly saturated across models, reinforcing that latency variability and cost efficiency are the primary differentiators in this evaluation.

**Figure 6:**
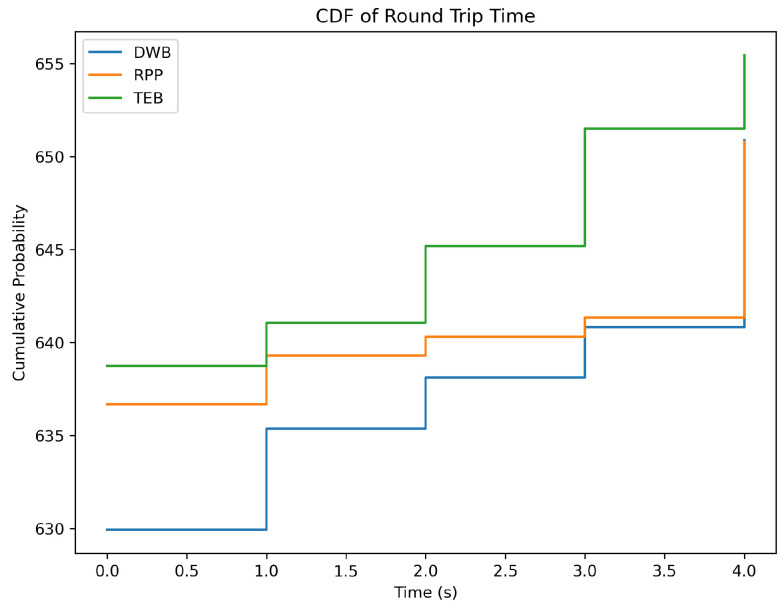
CDF of LLM round-trip latency, highlighting the variance between lightweight and heavyweight reasoning models.

Figure 6 further visualises the latency distribution using a cumulative density function (CDF). Faster models show steep curves with narrow variance, whereas slower models present long-tailed spreads.

These results confirm strong latency–reasoning trade-offs, with some LLMs unsuitable for real-time robotics despite high-quality reasoning performance.

### 3.3. LLM Parsing and Success Rate

**Figure 7:**
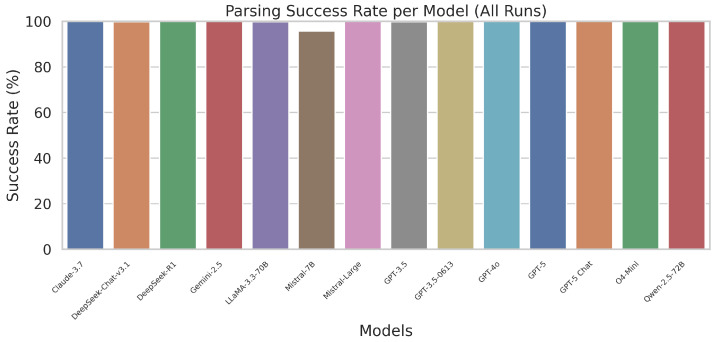
Success rate of all LLMs across the full command set.

The parsing success reflects the percentage of trials where an LLM produced valid JSON, formatted according to the required schema. Figure 7 presents the overall success rate for each model.

**Figure 8:**
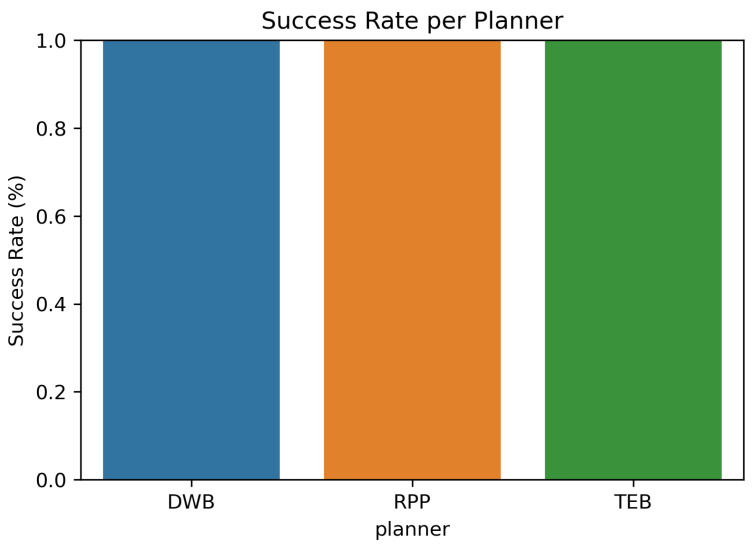
Parsing success rates by model across simple, moderate, and complex commands.

To provide a clearer comparison, Figure 8 displays a barplot of the parsing success across instruction complexities.

Lightweight models occasionally failed in complex instructions due to incomplete JSON outputs. All premium models maintained near-perfect success throughout.

### 3.4. Token Usage and Cost Efficiency

**Figure 9:**
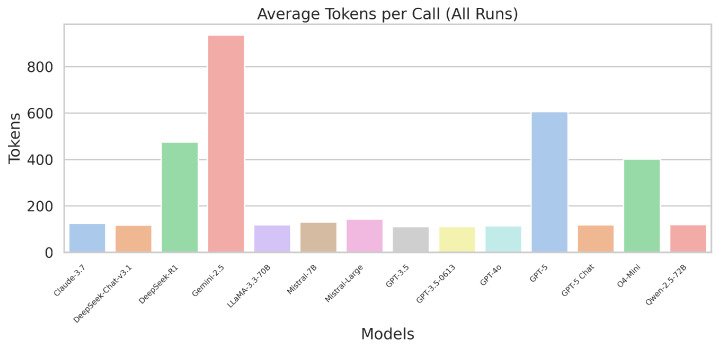
Average token usage per instruction for all LLMs.

Figure 9 shows the average number of tokens consumed per response. Models such as Gemini-2.5, GPT-5, and DeepSeek-R1 produced structured but long outputs, highlighting significantly higher operating costs compared to GPT-3.5, Claude, and LLaMA.

These findings emphasise the financial implications of deploying reasoning-heavy models for high-frequency robot interactions.

### 3.5. Planner Navigation Performance

The planner performance was measured in terms of the trajectory execution time, path smoothness, and consistency across repeated trials.

**Figure 10:**
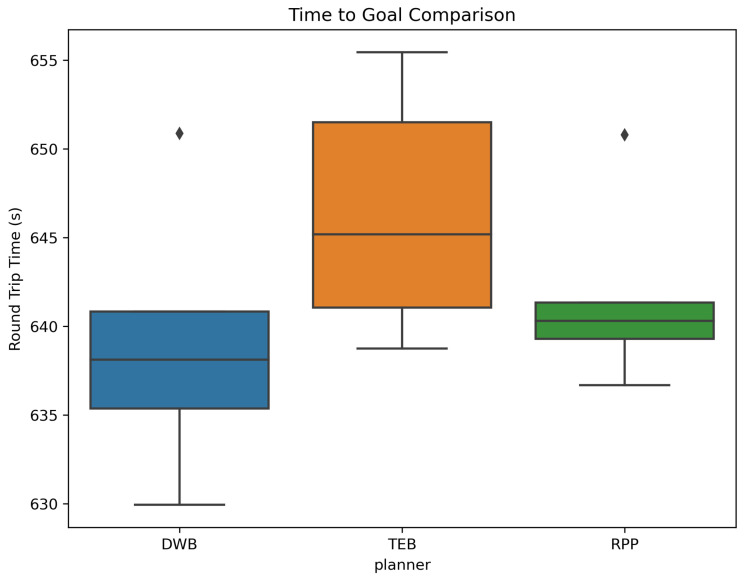
Execution time distribution for DWB, TEB, and RPP planners.

Figure 10 presents the distribution of navigation times for each local planner.

DWB achieved the shortest execution times but occasionally produced abrupt manoeuvres. TEB offered the smoothest and safest paths, but with higher computational overhead. RPP provided stable behaviour but struggled in cluttered scenarios.

**Figure 11:**
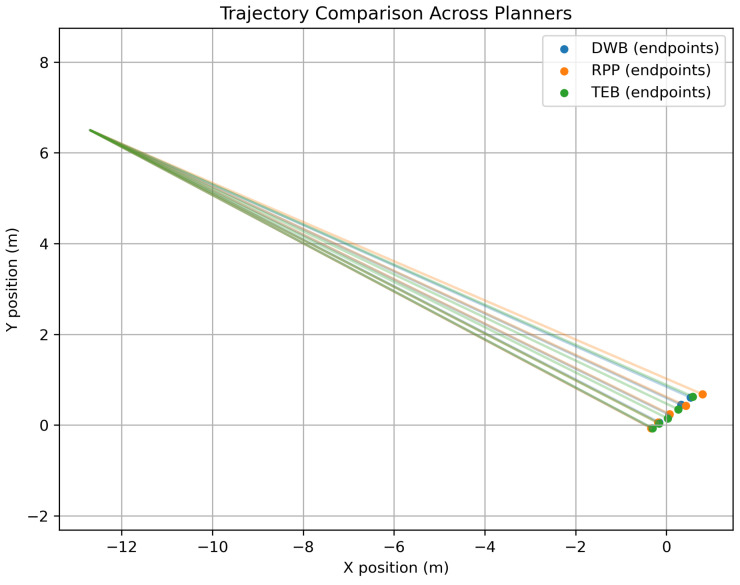
Representative navigation trajectories for each planner.

To complement the quantitative analysis, Figure 11 illustrates representative trajectories executed by each planner.

These results demonstrate clear performance trade-offs depending on the planner characteristics and scenario complexity.

### 3.6. Simulation Visualisation

**Figure 12:**
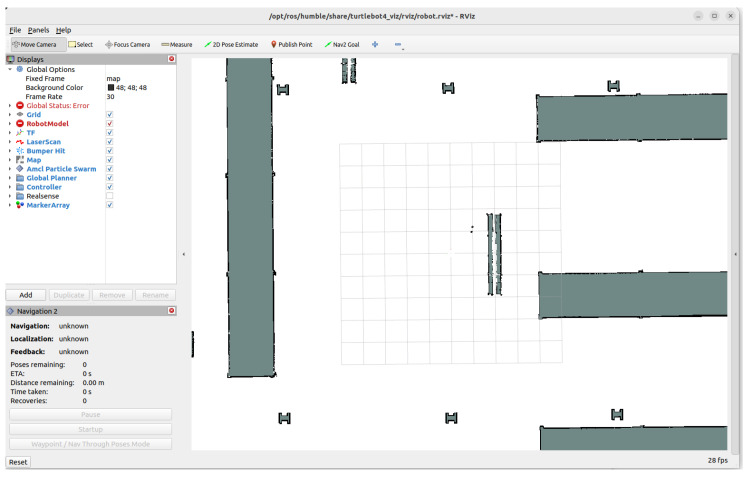
RViz 2 visualisation of the navigation goal and costmap layers.

**Figure 13:**
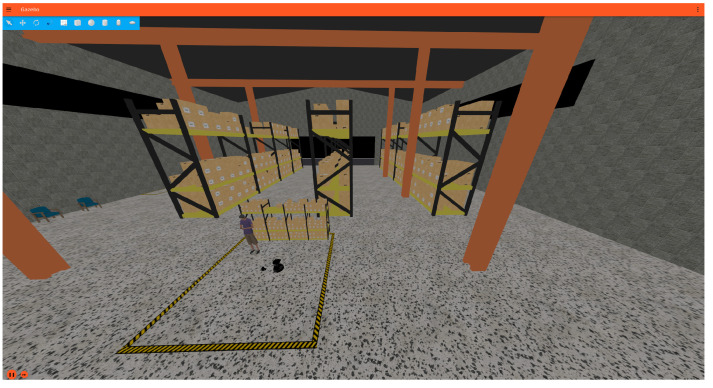
Gazebo simulation environment with TurtleBot4 navigating the scene.

Simulation outputs provide qualitative insight into system behaviour. Figure 12 shows the final goal representation in RViz 2, while Figure 13 shows the corresponding view in Gazebo Fortress.

The simulation visuals confirm that the pipeline successfully translated natural language commands into coherent navigation actions across planners and LLMs.

### 3.7. Consolidated LLM Performance Summary

Table 3 provides a unified comparison of the latency, success rate, and token usage.

In simple terms, lightweight LLMs delivered the fastest responses but occasionally struggled with complex instructions. Large reasoning models offered perfect parsing and stable behaviour, at the cost of significant latency and token consumption. Planner comparisons revealed that TEB produced the most reliable paths, DWB was the fastest, and RPP offered moderate stability with limitations in cluttered environments. These trends highlight the importance of selecting an LLM–planner pairing based on task demands, available computation, and acceptable latency for real-world robotic interaction.

## 4. Discussion

This section interprets the findings of the benchmarking experiments and interprets their broader implications for natural-language-guided robot navigation. While Section 3 presented detailed quantitative results, the present discussion analyses the underlying trends, trade-offs, and practical significance of the outcomes. The goal is to connect LLM performance, planner behaviour, and integrated pipeline dynamics to both theoretical understanding and real-world deployment considerations.

### 4.1. LLM Benchmarking Insights

#### 4.1.1. Latency and Responsiveness

Latency emerged as one of the most influential dimensions of LLM performance. Lightweight models such as GPT-3.5, GPT-4o, and Mistral-7B consistently produced sub-2-second response times with tight variability. These models are therefore well-suited to interactive robotics applications where command interpretation must occur promptly for safe operation. On the other hand, high-end reasoning-oriented systems like GPT-5, Gemini-2.5 Pro, and DeepSeek-R1 often took more than 10 s, and some even took more than 20 s. These models have advanced reasoning abilities, but their slow and often unreliable response times make them hard to use in situations where quick reactions are needed, like helping people with health problems or service robots that interact with people. CDF analysis reinforced this observation: models like GPT-3.5 and GPT-4o displayed steep compact curves, indicating predictable latency, whereas Gemini-2.5 Pro and DeepSeek-R1 showed broad dispersion. Predictability is as important as speed in HRI; users can adapt to a consistent delay, but unpredictable swings undermine trust and usability. It is also important to note that latency is not solely a function of the model. Network conditions, server load, and API congestion influenced the response times during testing. Periods of higher global server usage resulted in noticeable slowdowns. Future work may incorporate controlled 3G/4G/5G testing to measure environmental sensitivity. Nevertheless, the observed trends show that for real-time navigation tasks, latency-efficient models provide the most reliable interaction experience.

#### 4.1.2. Parsing Reliability

The parsing performance remained high among every model, and most LLMs produced almost 100% valid JSON results when prompted with schema constraints. This proves that sequential prompting, along with a robust schema validation and retry logic, can effectively turn natural-language commands into structured navigation objectives. Some smaller open-source algorithms sometimes gave outputs that were not correct, such as missing keys, extra descriptive text, or incorrect JSON syntax. This meant that parsing was successful about 95–97% of the time. However, these problems were mostly fixed by retrying and enforcing the schema. Parsing reliability in this controlled setting demonstrates that LLM-driven instruction parsing is feasible for robotics applications. However, generalisation to unconstrained human dialogue remains an open challenge. Real users may produce vague, ambiguous, or multi-turn instructions (e.g., “Can you grab that thing from over there?”). Small systems may have trouble without repeated clarification, but models like Claude-3.7 and GPT-4o may be more effective in handling ambiguity.

In brief, parsing success is an entry point metric: if an LLM fails to generate valid ordered outputs, no subsequent navigation can happen. The comprehension efficiencies that are almost perfect show that the present LLMs can be trusted to translate between human commands and robotic navigation processes when the prompts are controlled.

#### 4.1.3. Token Usage and Cost Considerations

The token efficiency varied substantially across models. Lightweight models such as GPT-3.5, LLaMA-70B, and Claude-3.7 typically consumed fewer than 150 tokens per command, while large reasoning models like GPT-5 or Gemini-2.5 Pro frequently exceeded 500–900 tokens. However, the token usage did not correlate directly with the cost. The true cost is shaped by the per-token pricing of each model, which varies considerably across providers. If the price per token is much higher, for example, a brief result from Gemini-2.5 Pro may cost more than an extensive output from GPT-3.5.

So, two important ideas come to light:

1. The number of tokens for an item is not a good way to guess its cost.
2. The predictability of how tokens will be used is often more important than how efficient they are.

Higher-end models not only used a lot tokens, but they also made things less predictable, sometimes returning extremely verbose responses and making the budget estimation complex. Lightweight models offered far more stable token patterns, making them easier to deploy at scale. In robotics deployments, where thousands of commands may be processed daily (e.g., warehouse fleets, hospitality robots), cost differences accumulate rapidly. For many practical cases, mid-tier or lightweight models offer the optimal balance of reliability, efficiency, and affordability.

### 4.2. Planner Benchmarking Insights

#### 4.2.1. Success Rates and Reliability

All planners, including DWB, RPP, and TEB, passed all tests with outstanding scores. This shows that Nav2 planners are very reliable when given clear goals in a controlled setting. This confirms that planner performance was not a limiting factor in the overall pipeline and establishes a solid baseline for LLM-driven navigation.

#### 4.2.2. Completion Time and Variability

Clear distinctions emerged in the round-trip time:

- **DWB:** fastest completion, low variability, and highly reactive.
- **RPP:** slightly slower but most consistent with the smallest variance.
- **TEB:** slowest average times, due to optimisation overhead.

This aligns with known characteristics of these planners. DWB prioritises responsiveness, RPP emphasises smooth tracking with stability, and TEB optimises trajectories using complex cost functions, sacrificing speed for comfort.

#### 4.2.3. Trajectory Characteristics

The trajectory analysis highlighted further contrasts:

- **TEB** produced the smoothest and most natural paths, ideal for human-interactive environments.
- **DWB** yielded segmented, sometimes abrupt movements typical of sampling-based local planning.
- **RPP** balanced stability and directness, offering simple and predictable motion.

These differences emphasise that the planner choice depends on the application context rather than a universal metric of “best performance”.

### 4.3. Trade-Off Analysis

The benchmarking results presented in the previous section demonstrate that while all LLMs and planners achieved high reliability, their behaviour varied significantly in terms of the latency, token usage, cost, and path characteristics. This section synthesises these findings and discusses the practical trade-offs that arise when deploying LLM-driven navigation systems.

#### 4.3.1. LLM Trade-Offs

The evaluated LLMs exhibited the highest variation in inference latency, cost, and token efficiency. Advanced reasoning models like GPT-5 and DeepSeek-R1 did a great job of interpreting tasks, but they had long round-trip times and used a lot of tokens. On the other hand, lightweight models like GPT-3.5 and Mistral-7B gave very quick responses, but they were not as reliable when given complicated instructions. Claude-3.7 and GPT-4o represented balanced mid-tier options, combining low latency with strong consistency.

Table 4 summarises these relationships.

#### 4.3.2. Planner Trade-Offs

Although all three Nav2 planners (DWB, TEB, RPP) achieved a 100% task completion rate, they differed in speed, variability, and motion behaviour. DWB produced the fastest runs and lowest variance, RPP offered the most consistent and predictable behaviour, while TEB generated smoother trajectories at the cost of increased computation time.

These differences are summarised in Table 5.

#### 4.3.3. Combined LLM + Planner Trade-Offs

In practice, system designers must jointly select both the LLM and planner. Different deployment scenarios benefit from different combinations. Table 6 provides the recommended pairings based on the latency requirements, hardware constraints, and application type.

## 5. Conclusions

This research introduced a latency-aware benchmarking framework that integrates multiple Large Language Models with the ROS 2 Navigation 2 stack to enable natural-language control of mobile robots. Using a simulated TurtleBot4 in Gazebo Fortress, the study the evaluated end-to-end latency, instruction-parsing reliability, trajectory quality, and task completion across a wide range of LLMs and Nav2 planners. The experimental results show that natural-language navigation is technically feasible and highly reliable across model families. Most LLMs translated free-form human instructions into actionable navigation intents with near-perfect success. The analysis also revealed consistent trade-offs between latency, reasoning ability, and computational cost. Small models like GPT-3.5 and Mistral-7B gave quick answers and used tokens well. On the other hand, larger models like GPT-5, DeepSeek-R1, and Gemini-2.5 Pro had better reasoning but took longer to make decisions and cost more to run. The way planners acted also had an effect on how well the whole system worked. DWB, RPP, and TEB all completed the task successfully, but they did so in different ways. DWB had the fastest turnaround time, RPP had very stable trajectories, and TEB had smoother paths but more variable execution. For a good balance, research settings that emphasise refined logic might choose GPT-4o or GPT-5 with DWB.

Our introduced system provides a replicable basis for assessing natural-language robotic navigation and offers pragmatic suggestions for the selection of LLMs and planners in accordance with performance limitations. Future research will expand the assessment to encompass physical robots, investigate multimodal language models, and formulate strategies to alleviate latency variability, thereby enhancing real-time natural language control in autonomous systems.

While this study focuses on a controlled simulation-based evaluation to enable fair and reproducible benchmarking, several important directions remain for future work. A natural next step is to extend the framework to physical robot deployments in order to assess the timing determinism, sensor noise, actuator delays, and safety-critical behaviour under real-world conditions. Such experiments would help validate how insights obtained from simulation transfer to embodied systems, where environmental uncertainty and hardware variability play a larger role.

In addition, future studies could extend this work to multi-robot settings, outdoor environments, and scenarios with constrained or intermittent network connectivity. Since cloud-based LLM inference is influenced by provider-side scheduling, server load, and network conditions, strict run-to-run determinism cannot always be ensured; carefully designed network-controlled experiments would therefore be valuable for better understanding the reproducibility limits in practical deployments. Another important direction is a more systematic investigation of structured-output retry mechanisms, including comparisons across different retry limits and an analysis of how these choices affect parsing success rates, particularly for smaller or less reliable LLMs. Such an investigation would provide stronger empirical or theoretical grounding for retry strategies and clarify their statistical impact on both robustness and latency. Beyond this, the result interpretability could be improved through alternative visualisations, richer annotations, or uncertainty-aware summaries that more clearly expose performance variability across models and planners. Support for multi-turn conversational interaction would further enhance the framework’s relevance to realistic human–robot interaction scenarios. Finally, integrating vision–language models (VLMs) represents a natural and promising extension, enabling joint evaluation of language understanding, perceptual grounding, and navigation in more visually complex environments [19,20,21]. Collectively, these directions would help narrow the gap between simulation-based benchmarking and sustained real-world deployment.

## Figures and Tables

**Table 1:** Representative parameter values for Nav2 local planners used in this study.

Planner	Key Parameters	Effect/Notes
RPP	lookahead_distance = 0.6 m	Higher values enable faster movement but reduce path smoothness; lower values improve stability at the cost of slower navigation.
TEB	weight_obstacle = 50.0	High values prioritise obstacle avoidance and safety but may produce longer detours; lower values allow shorter but riskier paths.
DWB	max_vel_x = 0.26 m/s, acc_lim_x = 2.5 m/s^2^	Defines the trade-off between speed and manoeuvrability. Higher values increase speed but reduce control in cluttered environments.

**Table 3:** Consolidated performance of LLMs across all runs. Values represent averages computed from the combined dataset.

Model	Latency (s)	Success Rate (%)	Avg. Tokens
Claude-3.7 Sonnet	2.79	100.0	125.1
DeepSeek-Chat v3.1	2.51	99.8	116.6
DeepSeek-R1	10.20	100.0	474.5
Gemini-2.5 Pro	10.50	100.0	935.6
LLaMA-3.3-70B Instruct	1.76	99.7	118.3
Mistral-7B Instruct	1.80	95.7	130.2
Mistral-Large	2.02	100.0	141.8
GPT-3.5	1.14	100.0	112.1
GPT-3.5-0613	1.30	100.0	112.6
GPT-4o	1.23	100.0	111.5
GPT-5	11.98	100.0	601.1
GPT-5 Chat	1.29	100.0	114.5
O4-Mini	6.34	100.0	402.1
Qwen-2.5-72B Instruct	2.07	100.0	125.3

**Table 4:** Trade-offs between LLMs for natural-language navigation.

Model	Latency	Success Rate	Token Efficiency	Relative Cost	Notes
GPT-5	High (∼12 s)	100%	Very high tokens	High	Best reasoning; slowest
GPT-5 Chat	Moderate (2 s)	100%	Low tokens	High	Stable and efficient
GPT-4o	Low (1 s)	100%	Low tokens	Moderate	Excellent balance of speed and accuracy
Claude-3.7	Moderate (2–3 s)	100%	Low tokens	Moderate	Reliable and interpretable
Gemini-2.5 Pro	High (>10 s)	100%	Very high tokens	High	Inconsistent latency
DeepSeek-R1	High (>10 s)	100%	High tokens	Low–Moderate	Reasoning-focused; slow
GPT-3.5	Very Low (<2 s)	100%	Very efficient	Low	Fastest and cheapest baseline
Mistral-7B	Very Low (<2 s)	95–100%	Efficient	Very Low	Ideal budget model

**Table 5:** Trade-offs between Nav2 planners.

Planner	Success Rate	Round-Trip Time	Variability	Path Characteristics	Notes
DWB	100%	Fastest (∼637 s)	Low	Reactive, segmented	Best speed and responsiveness
RPP	100%	Moderate (∼640 s)	Low	Balanced, stable	Reliable all-purpose planner
TEB	100%	Slowest (∼645 s)	High	Smooth trajectories	Best for human-facing comfort

**Table 6:** Recommended LLM and planner combinations for different deployment scenarios.

Scenario	LLM Choice	Planner Choice	Rationale
Research prototyping	GPT-4o	DWB	Fast iteration with reliable parsing and low latency
Human-facing assistive robots	Claude-3.7	TEB	Smooth paths and interpretable LLM outputs
Budget-constrained deployment	Mistral-7B or GPT-3.5	RPP	Low-cost LLMs paired with a stable, predictable planner

## Data Availability

Data supporting the findings of this study are available from the corresponding author upon reasonable request.
